# How Can We Reach You? An Overview of Establishing Effective Recruitment Strategies for Young Women Breast Cancer Survivors

**DOI:** 10.1097/cr9.0000000000000088

**Published:** 2026-08-07

**Authors:** Pearman D. Parker, Tiffany A. Bogan, Traci A. Owen, Reid D. Landes

**Affiliations:** College of Nursing, University of Arkansas for Medical Sciences, Little Rock, Arkansas (Parker and Bogan); Intimate Pathways Center for Sexual Health, Broken Arrow, Oklahoma (Owen); College of Medicine, University of Arkansas for Medical Sciences, Little Rock, Arkansas (Landes).

**Keywords:** Breast cancer survivorship, Recruitment, Sexual health, Social media, Young women

## Abstract

**Background:**

Young women (aged 18–50) breast cancer survivors often face troubling sexual side effects resulting from cancer and cancer treatment. Research interventions can target young women’s unique needs, but recruitment for this population remains a challenge.

**Objective:**

We sought to determine an effective strategy to recruit young women breast cancer survivors for our remote, nurse-led sexual health psychoeducational intervention EMBRACE (Empowerment through Mind and Body Reclamation After Cancer Experience).

**Methods:**

Our 5 major recruitment methods were: (1) using a study website; (2) distributing a digital study flyer; (3) placing physical rack cards in 2 nearby states; (4) employing snowball sampling through word-of-mouth; and (5) sharing Facebook posts.

**Results:**

Sixty-four young women breast cancer survivors expressed interest in being screened. They learned about our study through Facebook groups (n = 24; 38%), Facebook pages (n = 17; 27%), word-of-mouth (n = 16; 25%), Google searches (n = 4; 6%), and undisclosed searches (n = 3; 4%). Of the 64 women we screened, 55 met eligibility criteria, and 53 provided consent (96.4%). We withdrew 4 women who did not meet eligibility criteria, resulting in a sample of 49 women.

**Conclusions:**

Sharing directly with established cancer survivorship Facebook groups was the most effective recruitment strategy.

**Implications for Practice:**

Leveraging relationships using social media is the best method for recruiting young women breast cancer survivors. We recommend initiating contact with Facebook groups early in the development of the study.

**Foundational:**

Collaborating with partners on social media was the most effective recruitment method.

Young women (between ages 18 and 50) breast cancer survivors often face troubling sexual side effects resulting from cancer and cancer treatment.^1,2^ Decreased sexual functioning, disturbances in body image, and burdensome premature menopausal symptoms are commonly reported and can be especially detrimental to young women’s intimate relationships, overall quality of life, and well-being in survivorship.^3–5^ Along with these challenges, sexual health is often overlooked in survivorship care.^6^ As a result, interventions are being researched to specifically target these unmet clinical and relational sexual health needs.^7,8^ However, recruitment for this population remains a significant challenge.^9^

Young women frequently face time constraints from competing demands (ie, childcare concerns and career schedules), limiting their availability for participation in research and resulting in difficulties with recruitment.^10,11^ Additionally, young women breast cancer survivors are less likely to be in the clinic for frequent visits (compared to when they were in active treatment) and may not be aware of open clinical trials frequently promoted in the clinic.^12^ These less frequent clinic visits also reduce their exposure to traditional recruitment strategies used in clinic settings. The type of clinical trials may also be a factor related to recruitment. Behavioral interventions tend to be time-consuming and/or require participants to attend sessions in person.^13^ Geography can also be a barrier to recruitment and ultimately participation.^14^

Given these constraints, identifying useful recruitment strategies during pilot or feasibility trials can be extremely beneficial before scaling to a larger clinical trial, where one expects to recruit a much larger number of participants. Thus, we sought to evaluate and determine our most effective recruitment strategy for young women breast cancer survivors of our remote, nurse-led sexual health psychoeducational pilot intervention, Empowerment through Mind and Body Reclamation After Cancer Experience (EMBRACE). Our targeted recruitment goal for this feasibility trial was 42 women.

## Methods

### Study Aims and Design

Our study was a randomized clinical trial using a wait-list design to examine the feasibility (recruitment, retention, engagement, acceptability, and satisfaction) and preliminary efficacy of EMBRACE—a nurse-led psychoeducational sexual health intervention for young women breast cancer survivors. Our primary outcomes were sexual functioning, menopausal symptoms, and body image. The purposes of this paper are to describe and evaluate our most effective recruitment strategy for our pilot intervention, EMBRACE. The abstract of additional feasibility results (acceptability, satisfaction) is published elsewhere.^15^

Participants were randomly assigned to either (1) the immediate start group (and began the intervention immediately after enrolling) or (2) the delayed-start group (and began the intervention 16 weeks after enrolling). We used maximally-tolerated-imbalance randomization.^16^ Both groups received the intervention of 8 sessions lasting from 60 to 90 minutes every 2 weeks for the span of 16 weeks. The delayed-start group had an engaged waiting period of 16 weeks to serve as a control.^17^

All participants completed baseline surveys at least once, and the delayed-start group completed 2 sets of baseline surveys 16 weeks apart in accordance to wait-list design protocols.^17^ Once participants began the intervention, they completed postsession surveys on acceptability and applicability/satisfaction after each of the 8 sessions. We also collected postintervention surveys (separate from the session surveys) immediately after the eighth session and at 6-week follow-up. All surveys were administered electronically via REDCap.^18^

### Participant Eligibility

Participant inclusion criteria included: adult women diagnosed with breast cancer (stages I-III) between the ages of 18 and 50; currently between the ages of 18 and 50; completed active treatment (radiation/chemotherapy) within the last five years; taking hormonal therapies (i.e., aromatase inhibitors, tamoxifen); currently partnered. Our exclusion criteria included: breast cancer recurrence or metastatic breast cancer; women who lived outside of the nurse interventionist’s licensure coverage (i.e., Alaska, California, Hawaii, Illinois, Massachusetts, Michigan, Minnesota, Pennsylvania, Nevada, Oregon, Virgin Islands, American Samoa, or Commonwealth of Northern Marina Islands).^19^ We also excluded any participants who had no access to the internet or privacy, given the remote delivery and sensitive nature of topics within the intervention. Unfortunately, we also excluded anyone who could not read/speak English due to the limitations of our study team and nurse interventionist.

### Recruitment Strategies

Our five major recruitment methods were: (1) using a study website; (2) distributing a digital study flyer; (3) placing physical rack cards in 2 nearby states; (4) employing snowball sampling through word-of-mouth; and (5) sharing Facebook posts. For each of these methods, we relied on our team’s previously established community relationships to ask for endorsement, sharing, or support of our study.

### Description of Recruitment Content

#### Study Website

Before any distribution of study promotion materials, we established our EMBRACE study website. The website was housed through the University of Arkansas for Medical Sciences Cancer Institute Blog, which promoted clinical trials. The design was an interlay of complementary graphics and images with short, bulleted points highlighting eligibility criteria and incentives ($150 in gift cards, vaginal dilator, vaginal moisturizer, and lubrication products) as well as private one-on-one sessions with our nurse interventionist. We also featured our nurse interventionist’s role as an oncology sexual health specialist registered nurse. An “apply now” link was embedded on the website where interested women could fill out their contact information to be screened by the study team, preceding consent discussion.

#### Digital Study Flyer

We worked with a graphic artist to create our digital recruitment flyer and maintained the same color scheme as our study logo. The flyer included pertinent study information (ie, eligibility criteria, incentives) that followed readability guidelines in a welcoming font. We also placed a QR code linking to the online contact information form (described above) for women to complete if they were interested in setting up a screening call. The digital study flyer was shared via e-mail to 7 clinical oncology groups with an accompanying recruitment announcement. The groups were encouraged to share with patients and caregivers at their discretion. See the Supplementary Appendix, https://links.lww.com/CR9/A19 for the digital study flyer.

#### Physical Rack Cards

We also made 8.75 × 4.25” rack cards, which were similarly designed to our study flyer and contained the same pertinent study information (ie, eligibility criteria and incentives). The cards also included the QR code linking to the online contact information form. Each card was printed on high-gloss card stock. We placed the cards at 6 local oncology and primary care clinics and support groups in Oklahoma and Louisiana. We leveraged our previous clinical partnerships to secure support and agreement to share our rack cards. Between 30 and 50 cards were distributed at each location.

#### Snowball Sampling Through Word-of-Mouth

We also encouraged potential participants to share study information with friends, colleagues, and anyone in their peer and social networks of choice by word-of-mouth as an informal snowball sampling strategy.

#### Facebook Posts

Most of our time dedicated to recruitment efforts was focused on creating content and images used for posts in population-relevant Facebook groups. We wrote 4 main posts, which were shared across various Facebook groups and pages. Our posts were made public, allowing users to freely share the posts without restriction. Our posts were written with compelling, positive verbiage, and we used graphics of our study logo and stock photo images. Each post included a link to the study website as described above. Once the posts were created and allowed to be shared, our time commitment drastically decreased as the sharing was done freely from Facebook group users without our oversight.

All of our recruitment materials were approved through the University of Arkansas for Medical Sciences Institutional Review Board Protocol #276879.

### Flow of Recruitment

After potential participants learned about the study, they chose to complete an online contact information form (routed from the QR code or direct link on the study website) housed in REDCap^18^ to be contacted by our study team. To track recruitment strategies, potential participants were asked in the contact information form how they learned about the study by selecting from a list of our recruitment sources. Participants who selected “social media” were prompted to specify the social media platform where they learned about the study, while those who selected “flyer” were prompted to provide the location where they saw the flyer. Our research assistant set up screening calls, further described the study, explained the randomization process, and proceeded with a consent discussion. Additionally, the screening calls were usually scheduled during off-hours to best meet the needs of the potential participants. Once we obtained consent, we randomized them into either the immediate start or delayed-start groups. We then shared their information with our clinical partner, who scheduled their first session depending on the group assignment. All of our screening and consenting efforts were set up on a continuous basis.

### Analysis

We imported data from REDCap^18^ into SPSS, where we ran descriptive statistics to calculate the frequencies of recruitment methods cited by participants most often. We continued to use descriptive statistics to calculate the means and frequencies of the demographics and cancer characteristics of our sample. All analyses were conducted in IBM SPSS Statistics, version 31.^20^

## Results

Recruitment opened in mid-October 2024 and closed in early February 2025. One hundred and ninety-three women expressed an interest in being contacted for screening. We attempted to contact the first 90 women who completed the contact interest form. We were unable to reach 26 of these women. Given our study-end sample size goal of 42 and continuous enrollment design, we achieved our sample size goal after completing 64 screens and subsequently closed recruitment. We successfully screened about two-thirds of the 90 women we attempted to contact.

Most of the 64 young women we screened learned about our study through Facebook posts within Facebook groups (*n* = 24; 38%), through business or individual Facebook pages (*n* = 17; 27%), by word-of-mouth (*n* = 16; 25%), Google searches (*n* = 4; 6%), and undisclosed sources (*n* = 3; 4%). Two women reported they learned about the study through ‘direct mailings,’ but we did not distribute any mailings and grouped them as ‘undisclosed sources.’ We also had no reports of women learning about the study through flyers or placement of rack cards. Of the 64 women we screened, 55 met eligibility criteria, and 53 provided a signed consent (96%). One scammer was identified before randomization and administratively withdrawn (identification of scammers is discussed later in the text), resulting in 52 participants who were randomized. We considered a person recruited when we received a signed consent.^21,22^ Our recruitment rate was 13 per month over the course of 4 months.

Following randomization and before beginning any sessions, 2 additional scammers were identified and removed. One participant was also withdrawn due to state eligibility restrictions but did not complete baseline assessments. Thus, we did not include the 3 in the subsequent sample statistical summaries. The statistical summaries presented below are of the sample consisting of 49 participants (Figure 1).

Of the 49 legitimate participants who provided consent, their ages ranged between 30 and 50 (mean = 42.8, SD = 4.8); most were married or living with their partner (n = 45, 92%) and were at least college graduates *(n* = 41, 84%). Additionally, the women in our study were mostly employed full-time (n = 32; 65%) with yearly household incomes of greater than $110,000 (n = 26, 53%) (Table 1). They were mostly residents in the Midsouthern region of the United States of Oklahoma, Missouri, Texas, and Louisiana (n = 25, 51%) (Table 2). Breast cancer characteristics were varied, but most of the women were either Stage 1 (n = 17, 35%) or Stage 2 (n = 21, 43%), with variations in subtype (Table 3). Lastly, an overwhelming number of participants were on hormone therapy (n = 44, 90%) and almost all received chemotherapy (n = 37, 76%), mastectomies (n = 37, 76%), and/or radiation (n = 33, 67%) (Table 4).

### Unexpected Spike in Completed Contact Screener Forms

Each month, an average of 15 young women completed the contact screener form to be screened between October 2024 and December 2024. However, we saw a spike in completed contact screener forms in the middle of January 2025 after our nurse interventionist promoted the study to the Facebook group, sex after breast cancer, which has more than 15,000 members. Our nurse interventionist frequently posted educational content in this group for the past 6 years and was well known in the Facebook group. She gave an unrelated sexual health educational lecture via Facebook Live and made a separate postpromoting the study. Within 24 hours of making the separate postpromoting the study, more than 130 women filled out the contact form for our study (Figure 2). This unexpected spike in potential participants allowed us to increase our screening rate. Without the spike, we may have had to wait an additional 3 months (15 completed forms per month) to have accumulated enough contacts to screen and enroll participants to meet our enrollment goal of 42. Our website traffic reflected the same sharp increase; after our nurse interventionist’s post on sex after breast cancer, our page received more than 800 views in January. Before the January post, our website page received an average of 225 views per month (Figure 2).

### Identification of Scammers

Given the largely online nature of our recruitment efforts, we were vulnerable to scammers or professional research participants who complete online studies for electronic gift cards.^23^ We identified 1 scammer before consent and 2 after randomization but before starting the sessions. We noticed that scammers used uncommon tactics. First, multiple contact screener forms were completed at the same time. Second, possible participants requested to have the screening calls with our Research Assistants via Zoom without their cameras turned on instead of a phone call. Third, possible participants reported no physical address (or we could not verify that an address was legitimate despite searching), which was necessary for mailing the intervention materials. Fourth, these women had little to no emotion during the screening calls and had variations in speech with audio interference. Fifth, these possible participants were only interested in physical gift cards and did not want to receive any electronic gift cards, as noted within our protocol. Once we identified these behaviors, we submitted a revision to the protocol to include Zoom-based screening calls with a camera. The new process required potential participants to use a camera before proceeding with the consent discussion. The protocol change allowed us to better identify any potential scammers.

## Discussion

We successfully met our recruitment goal of the feasibility trial, EMBRACE, averaging 13 enrolled participants each month over the course of 4 months. We exceeded our recruitment goal, but our sample was largely homogeneous in terms of marital status, education, income, and employment. This may have been largely due to our focus on using social media as a major avenue for recruitment. Valle and colleagues found similar skewedness (mostly women and those with higher education) in their sample when using social media recruitment. However, the authors were still able to reach their recruitment goals of adolescent and young adult cancer survivors through unpaid social media outreach. Additionally, they were able to recruit a nationwide sample given accessibility for their mHealth intervention.^24^ Whereas our sample was largely from the Midsouthern region, we still were able to have a nationwide reach within the limits of our nurse interventionist’s licensure.^19^ Our homogeneous sample may also have been the result of only using Facebook pages and groups with website promotion as methods to recruit. We did not access various social media channels (ie, Reddit, X, and Instagram) due to institutional limitations. Mizrach and colleagues expanded their search beyond Facebook and found more diversity in their sample of 72 adolescent and young adult cancer survivors (to which some of our participants would be categorized as young adults). In fact, 61% of their 72 participants were recruited through social media (n = 44): namely, 40% from Reddit (n = 29), 18% from Facebook (n = 13), and 3% from Twitter (n = 2). Their approach yielded a more diverse sample that included survivors who most recently completed treatment, were more geographically diverse, and were otherwise underrepresented.^25^ However, the authors also found similar challenges with scammers as we did.^25^

We were surprised at the presence of scammers given our involved screening process for a 16-week psychoeducational sexual health intervention. Most of the scammers identified in the literature were survey-based designs^23,26^ and some qualitative studies,^27,28^ but we found little evidence of scammers infiltrating time-intensive behavioral interventions, even with online recruitment methods. Despite the seemingly protective nature of our 16-week trial, we still identified scammers after our study opened. We were able to place safeguards in our screening process to prevent or identify scammers by implementing a requirement to turn on a camera during the video calls. However, adding safeguards before consenting may be beneficial for streamlining screening calls. Bell and colleagues^29^ provided clear guidelines on how to best mitigate scammers, including, but not limited to, monitoring for sequential attempts from the same IP address, considering withholding advertisements of monetary compensation for study enrollment, and automatically excluding responses with SPAM e-mails. These recommendations could be useful even for time-intensive behavioral interventions and save time on the screening process with study team members.

Despite navigating around and protecting against scammers, we found social media to be the most effective recruitment method above in-person methods (ie, sharing of rack cards and flyers). While we suspected social media would be the most effective (and thus our recruitment efforts were largely online), we did not anticipate the unexpected surge in contact screener forms following an educational guest lecture and subsequent post to the sex after breast cancer Facebook group from our nurse interventionist. Young women breast cancer survivors often rely on social media as a means of support, education, and resources,^30^ but we did not anticipate such an overwhelming response to our study. However, we believe this surge was due to our nurse interventionist’s previously established, longstanding relationship within the Facebook group. Even though this recruitment method used social media, the importance of cultivating and maintaining relationships with the intended population is a longstanding, effective recruitment method most frequently appearing in community-based participatory research.^31,32^ Similarly, Weiner and colleagues^33^ employed a community-based participatory research approach to recruit young breast cancer survivors through social media for a physical activity intervention. Their Haus of Volta Community Advisory Board recommended social media recruitment efforts to be focused on organizations like Young Survival Coalition and Living Beyond Breast Cancer. The efforts bridging relationships and social media were successful; more than 76% of their participants were recruited through social media.^33^ While our nurse interventionist was not on an advisory board for the EMBRACE study, she fulfilled the role of a community advocate and educator, which most likely generated an additional level of trust and enthusiasm for our study, contributing to the overwhelming positive response in the surge of contact screener forms.

## Implications for Practice

We believe our findings have significant implications for practice. Facebook groups specific to populations of interest may be an ideal resource for recruitment for research studies. However, promoting studies within these groups is best accomplished through preexisting relationships. We recommend that researchers thoroughly assess the partnerships within the study team to determine any preexisting relationships with Facebook groups or in the community. If there are no established relationships, we recommend initiating contact with Facebook groups or communities early in the development of the study design. Further, acknowledging recruitment partnerships and returning data to such groups (ie, sharing publications or findings within the Facebook groups) would establish reciprocity^34^ regardless of the origins of the partnerships. Additionally, we believe our practical methods (ie, study team members screening during off hours, making appointments in advance to adjust for time zone differences) for screening potential participants may have led to successful recruitment methods.

## Limitations

The recruitment methods we used were successful overall, and we met our recruitment goals in just a few months. However, despite the success of our strategies, our study is not without limitations. First, we focused heavily on social media as a method to have the farthest reach. However, we may have inadvertently excluded young women who were not present on social media or who were less active online. Second, we placed rack cards in the Midsouth states of Oklahoma and Louisiana. Consequently, we may have oversampled from these states despite our national reach using social media. Third, the contact interest form completed by interested participants did not include demographic data. Thus, we could not better describe our overall sample of those who expressed interest (N = 193) or statistically test for differences between recruitment methods. Fourth, due to an overlooked mistake in our data collection forms, we did not obtain racial demographic data from participants and thus could not draw conclusions about recruitment methods affecting the diversity of our sample. Fifth, we did not obtain any demographic data on the partners of our participants (ie, sexual orientation) and cannot make conclusions regarding the sexual diversity of our sample.

## Conclusion

Strategically using social media serves as a practical and effective recruitment method for breast cancer survivorship studies, especially when sharing information with Facebook groups, pages, and websites. However, the most impactful methods involve leveraging preexisting relationships within these online groups and communities on social media. Maintaining these relationships, acknowledging their support, and disseminating results back to such groups can lead to well-established, strong, mutually beneficial partnerships between researchers, community organizations, and participants. Ultimately, these digital recruitment strategies can help ensure that breast cancer survivorship research is more inclusive and responsive to the needs of the young women breast cancer survivorship community.

## Supplementary Material

Supplementary Material

Supplemental digital content for this article is available at https://links.lww.com/CR9/A19.

## Figures and Tables

**Figure 1.**
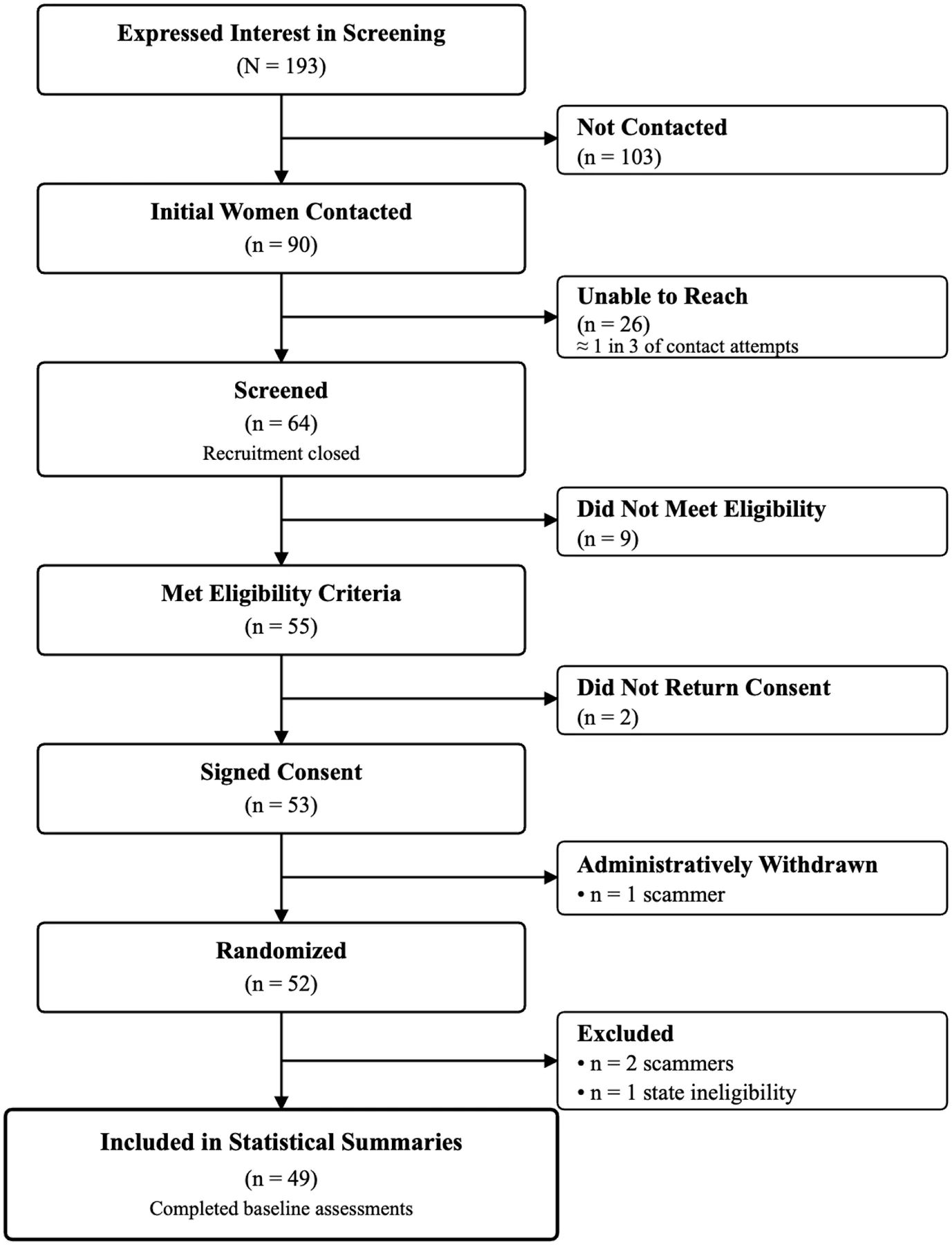
Recruitment Flowchart

**Figure 2.**
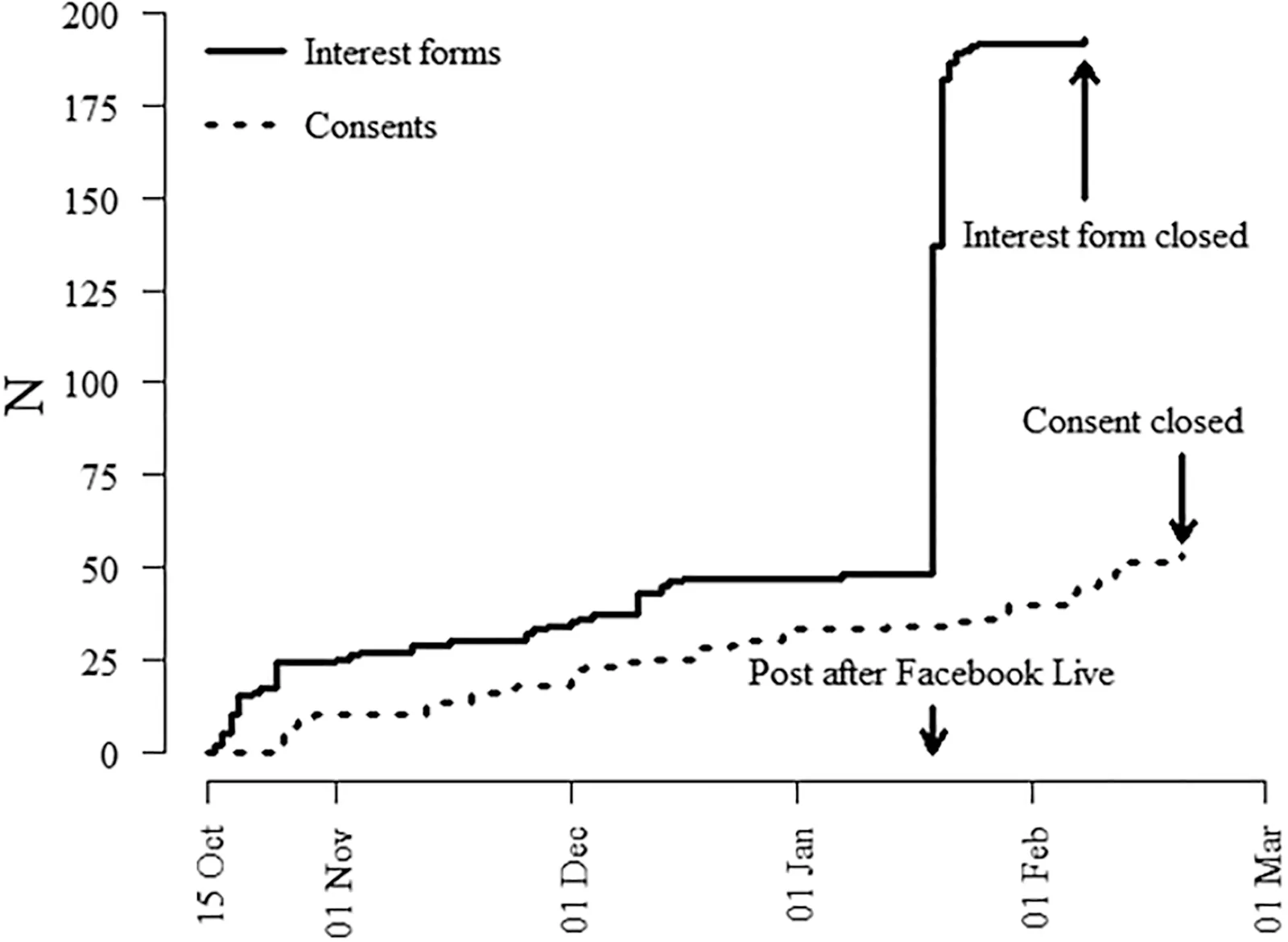
Timeline of interest forms and consents completed.

**Table 1.** Demographics

	Total (N = 49)	
Variables	n	%
Ethnicity		
Hispanic	7	14
Non-Hispanic	41	84
Unknown	1	2
Marital status		
Not married	2	4
Single	1	2
Living together	3	6
Married	42	86
Divorced/separated	1	2
Education		
High school diploma	4	8
Vocational training	4	8
College graduate	22	45
Postgraduate work	19	39
Household income		
<$30,000	1	2
$30,000–$49,999	6	12
$50,000–$69,999	5	10
$70,000–$89,999	6	12
$90,000–$109,999	4	8
>$110,000	26	53
Prefer not to answer	1	2
Employment		
Full-time	32	65
Part-time	11	22
Disabled	1	2
Housewife/stay-at-home mother	3	6
Student	1	2
Other	1	2

Age during data collection: Range = 30–50, mean = 42.8, SD = 4.8.

**Table 2.** Breakdown of Participants from Each State

State	n
OK	11
TX	6
LA	4
MO	4
OH	4
GA	3
KS	2
NC	2
NY	2
FL	2
IL	2
IA	1
AR	1
CA	1
ID	1
NJ	1
SC	1
WI	1
Total *N* =	49

**Table 3.** Overview of Breast Cancer Stages and Substages

	Total (N = 49)	
	n	%
Stage 1	9	18
1A	3	6
1B	5	10
Stage 2	9	18
2A	5	10
2B	7	14
Stage 3	3	6
3A	1	2
3B	2	4
3C	2	4
Unspecified	3	6

Breast cancer stages were self-reported; some participants clarified substage, and others only reported the broader stage.

**Table 4.** Frequencies of Histories of Cancer Treatments

	Total (N = 49)	
	n	%
Hormone therapy	44	90
Chemotherapy	37	76
Mastectomy	37	76
Radiation	33	67
Lumpectomy	14	29
Herceptin	10	20
Immunotherapy	5	10
Other	15	31

Hormone therapy was an inclusion criterion; we did not verify against medical records. All recruited participants passed the screening question of taking hormone therapy. Percentages do not add to 100% because some participants received more than one therapy.
