# Supplementary material for: How Can We Reach You? An Overview of Establishing Effective Recruitment Strategies for Young Women Breast Cancer Survivors
Source: Cancer Care Res Online. Author manuscript; Available in PMC 2026 Oct 8. (PMC13645383; doi:10.1097/cr9.0000000000000088)
Supplement: Supplementary file 1 [file NIHMS2213781-supplement-Supplementary_Material.pdf]

PARTICIPANTS NEEDED FOR STUDY THAT MAY

IMPROVE SEXUAL HEALTH

IN BREAST CANCER SURVIVORSHIP

If eligible, you will participate in a 16-week intervention with a sexuality-certified registered nurse.

ARE YOU ELIGIBLE?

- Young woman between the ages 18 – 50
- Diagnosed with breast cancer between 18 – 50
- Completed active treatment (chemotherapy, radiation) in the last five years
- Partnered
- Currently taking hormonal therapies (*i.e., tamoxifen or aromatase inhibitors*)
- No history of breast cancer recurrence

YOU WILL RECEIVE

- Receive $150 in gift cards
- Products and educational materials
  - Vaginal dilator
  - Vaginal rehydration

GET IN TOUCH

If interested, please scan the QR Code, email, or call the number provided, so we can schedule a call.

Principal Investigator
Pearman Parker, PhD, MPH, RN, PMH-BC
pparker@uams.edu

Research Assistant
Brooklyn Tody, BS
EMBRACE@uams.edu

501.779.8984

UAMS Winthrop P. Rockefeller Cancer Institute

UAMS College of Nursing

embrace

PARTICIPANTS NEEDED FOR A STUDY THAT MAY

IMPROVE SEXUAL WELLNESS

IN BREAST CANCER SURVIVORSHIP

If eligible, you will participate in a 16-week intervention with a sexuality-certified registered nurse.

ARE YOU ELIGIBLE?

- Young woman between the ages 18 – 50
- Diagnosed with breast cancer between 18 - 50
- Completed active treatment (chemotherapy, radiation) in the last five years
- Partnered
- Currently taking hormonal therapies (*i.e., tamoxifen or aromatase inhibitors*)
- No history of breast cancer recurrence

YOU WILL RECEIVE

- Receive $150 in gift cards
- Products and educational materials
  - Vaginal dilator
  - Vaginal rehydration

GET IN TOUCH

If interested, please scan the QR Code, email, or call the number provided, so we can schedule a call.

Principal Investigator
Pearman Parker, PhD, MPH, RN, PMH-BC
pparker@uams.edu

Research Assistant
Brooklyn Tody, BS
EMBRACE@uams.edu
501.779.8984

UAMS Winthrop P. Rockefeller Cancer Institute

UAMS College of Nursing

embrace
